# The Gut Microbiota of Critically Ill Patients With COVID-19

**DOI:** 10.3389/fcimb.2021.670424

**Published:** 2021-06-29

**Authors:** Paolo Gaibani, Federica D’Amico, Michele Bartoletti, Donatella Lombardo, Simone Rampelli, Giacomo Fornaro, Simona Coladonato, Antonio Siniscalchi, Maria Carla Re, Pierluigi Viale, Patrizia Brigidi, Silvia Turroni, Maddalena Giannella

**Affiliations:** ^1^ Microbiology Unit, Department of Experimental, Diagnostic and Specialty Medicine, IRCCS St. Orsola Hospital and University of Bologna, Bologna, Italy; ^2^ Department of Pharmacy and Biotechnology, University of Bologna, Bologna, Italy; ^3^ Department of Medical and Surgical Sciences, University of Bologna, Bologna, Italy; ^4^ Infectious Diseases Unit, Department of Medical and Surgical Sciences, IRCCS St. Orsola Hospital, Bologna, Italy; ^5^ Intensive Care Unit, Department of Medical and Surgical Sciences, IRCCS St. Orsola Hospital and University of Bologna, Bologna, Italy

**Keywords:** SARS-CoV-2, COVID-19, gut microbiota, intensive care unit, bloodstream infection

## Abstract

The SARS-CoV-2-associated COVID-19 pandemic has shaken the global healthcare system. Although the best-known symptoms are dry cough and pneumonia, viral RNA has been detected in the stool and about half of COVID-19 patients exhibit gastrointestinal upset. In this scenario, special attention is being paid to the possible role of the gut microbiota (GM). Fecal samples from 69 COVID-19 patients from three different hospitals of Bologna (Italy) were analyzed by 16S rRNA gene-based sequencing. The GM profile was compared with the publicly available one of healthy age- and gender-matched Italians, as well as with that of other critically ill non-COVID-19 patients. The GM of COVID-19 patients appeared severely dysbiotic, with reduced diversity, loss of health-associated microorganisms and enrichment of potential pathogens, particularly *Enterococcus*. This genus was far overrepresented in patients developing bloodstream infections (BSI) and admitted to the intensive care unit, while almost absent in other critically ill non-COVID-19 patients. Interestingly, the percentage of patients with BSI due to *Enterococcus* spp. was significantly higher during the COVID-19 pandemic than in the previous 3 years. Monitoring the GM of critically ill COVID-19 patients could help clinical management, by predicting the onset of medical complications such as difficult-to-treat secondary infections.

## Introduction

Severe acute respiratory syndrome coronavirus 2 (SARS-CoV-2)-associated coronavirus disease 2019 (COVID-19) has gripped the world in a pandemic, challenging its culture, economy and healthcare infrastructure (Huang et al., 2020; Zhu et al., 2020). Although it is primarily considered an influenza-like respiratory disease with a global mortality rate of 2.2% (WHO, 2020), SARS-CoV-2 RNA has also been detected in the stools of infected patients causing severe gastrointestinal symptoms, such as diarrhea, nausea, vomiting and abdominal pain, in up to 1 in 5 infected patients (Cheung et al., 2020; Tariq et al., 2020; Wan et al., 2020). Surveys from Wuhan (China), where the outbreak began, showed that 23% of COVID-19 patients had only gastrointestinal symptoms, 33% of patients had both respiratory and gastrointestinal problems while the remaining 44% were only respiratory ill (Han et al., 2020; Scaldaferri et al., 2020). This is due to the ability of SARS-CoV-2 to enter human cells *via* the ACE2 (angiotensin-converting enzyme 2) receptor, which is highly expressed also on ileal and colonic cells (Lamers et al., 2020), thus leading to enteric manifestations through virus-induced immune-mediated damage (Guan et al., 2020; Huang et al., 2020; Wan et al., 2020; Zhu et al., 2020). As expected, the loss of intestinal homeostasis resulting from viral infection does not spare the gut microbiota (GM), *i.e.*, the complex microbial community hosted in our intestine, universally recognized as a key element for host physiology (Gilbert et al., 2018; Turroni et al., 2018). The first reports in small cohorts of COVID-19 patients from China documented the presence of a dysbiotic state, also affecting the fungal component (Gu et al., 2020; Zuo et al., 2020a; Zuo et al.,2020b; Chen et al., 2021; Zuo et al., 2021). This dysbiosis is featured by reduced diversity, depletion of beneficial commensals, mainly short-chain fatty acid (SCFA) producers from *Lachnospiraceae* and *Ruminococcaceae* families, and enrichment in opportunistic pathogens or pathobionts. Interestingly, these alterations appear overall distinct from other viral infections as H1N1 (Gu et al., 2020), more severe in patients with high SARS-CoV-2 infectivity (Zuo et al., 2021), and partly persist over time, particularly low richness, even long after viral clearance and symptom resolution (Zuo et al., 2020a; Chen et al., 2021). These microbial signatures are likely to contribute to further impaired immune responses, even through the gut-lung axis, with breakdown of mucosal barriers and translocation of microbial components or microbes themselves, thus potentially fueling systemic hyperinflammation, as found in patients at risk of fatal outcome (Chen et al., 2020). In this regard, it is worth mentioning that Zhou et al. (2020) reported that half of non-surviving COVID-19 patients experienced secondary bacterial and fungal infections. Although these estimates do not appear to be confirmed worldwide (Rawson et al., 2021), secondary invasion by opportunistic pathogens is recognized as critically important during the disease course, thus requiring careful monitoring for improved antimicrobial stewardship (Cox et al., 2020).

In an attempt to further extend this knowledge, here we characterized, by high-throughput 16S rRNA gene sequencing, the GM of 69 COVID-19 patients from three different hospitals from the metropolitan area of Bologna, in Emilia Romagna region (Italy), one of the most affected by the pandemic. Fecal samples were collected in the midst of the COVID-19 outbreak, from April to May 2020. GM profiles were compared with publicly available profiles of healthy age- and gender-matched Italians, as well as those of other critically ill non-COVID-19 patients.

## Materials and Methods

### Study Design

The study was approved by the Ethics Committee of the promoting center (Comitato Etico Indipendente di Area Vasta Emilia Centro, n. 283/2020/Oss/AOUBo).

We prospectively enrolled consecutive adult (≥18 years) patients diagnosed of SARS-CoV-2 virus infection, hospitalized from April 21 to May 9, 2020, in two teaching hospitals (St. Orsola and Bellaria Hospital) and one tertiary non-teaching hospital (Maggiore Hospital) from the metropolitan area of Bologna (Italy). Subjects were excluded if they had not a laboratory confirmed diagnosis of SARS-CoV-2 infection, and/or clinical data were unavailable. Underlying conditions were recorded according to Charlson comorbidity index (Charlson et al., 1987). As for SARS-CoV-2 infection, we collected: i) date of symptoms onset; ii) date and symptoms at hospitalization; iii) vital signs, laboratory tests and imaging findings at hospitalization; iv) clinical severity at hospitalization classified according to sequential organ failure assessment; and v) all administered antiviral and immunomodulatory treatments. Admission to ICU, management of respiratory failure, renal replacement therapy and inotropic support, as well as don’t resuscitate order established by attending physician were recorded. Occurrence of bacterial superinfection was assessed according to Centers for Disease Control and Prevention (CDC) criteria (Horan et al., 2008). A fecal sample was collected at the time of infectious disease consultation in a sterile plastic container and kept at -80°C until further processing.

### SARS-CoV-2 Diagnosis

Microbiological diagnosis of SARS-CoV-2 infection was performed by detection of SARS-CoV-2 RNA in respiratory samples (oropharyngeal-nasopharyngeal swab, bronchoalveolar lavage or broncoaspirate), as previously described (Bartoletti et al., 2020). Briefly, total DNA/RNA was extracted from samples by QiaSymphony (QIAGEN) and detection of SARS-CoV-2 was performed by real-time RT-PCR targeting regions in the N gene following the US CDC protocol.

### Microbial DNA Extraction, Library Preparation, and Sequencing

All fecal samples were processed in a biosafety level 3 laboratory [Centro di riferimento regionale per le emergenze microbiologiche (CRREM), St. Orsola Hospital, Bologna, Italy]. Microbial DNA was extracted using the repeated bead-beating plus column method (Yu and Morrison, 2004), with slight modifications as previously described (Turroni et al., 2017). For library preparation, the V3-V4 hypervariable region of the 16S rRNA gene was amplified by using the 341F and 785R primers (D’Amico et al., 2019). The final libraries, indexed and purified, were sequenced on an Illumina MiSeq platform, with a 2 × 250 bp paired-end protocol according to the manufacturer’s instructions (Illumina). Sequencing reads were deposited in the National Center for Biotechnology Information Sequence Read Archive (NCBI SRA; BioProject ID PRJNA700830). Further details are available in the **Supplementary Methods**.

### Bioinformatics and Statistics

Raw sequences were processed using a combined pipeline of PANDASeq (Masella et al., 2012) and QIIME 2 (Bolyen et al., 2019). Length and quality-filtered reads were binned into amplicon sequence variants (ASVs) using DADA2 (Callahan et al., 2016). Taxonomic assignment was performed using VSEARCH (Rognes et al., 2016) against the Greengenes database (May 2013 release). Chimeras were discarded during analysis. Publicly available 16S rRNA gene sequences of 69 healthy subjects matched by age [median, IQR (days): 59, 48-95], sex (39 females, 30 males) and geography (across Italy) were downloaded from databases and processed as above [33 subjects: NCBI SRA, Bioproject ID SRP042234 (De Filippis et al., 2016); 36 subjects: MG-RAST ID mgp17761 (Biagi et al., 2016)]. GM sequences of 16 patients admitted to ICU at St. Orsola Hospital after undergoing liver transplantation in October 2019-February 2020, were also used for comparative purposes (unpublished data). We selected non-COVID-19 patients who were best matched for known microbiota-associated confounding factors (*i.e.*, age [median, IQR (years): 57, 47.8-63] and sex ratio [50%]) as well as for other therapy and hospitalization-related confounders (*i.e.*, exposure to antibiotics in the 2 weeks prior to fecal sampling [69%], time interval between hospitalization and sampling [median, IQR (days): 12, 8-15.5], and development of bacterial infection after sampling [12.5%]). All fecal samples had been collected by the authors and processed in the same laboratory, then subjected to the same wet and *in silico* analysis steps. Alpha diversity was calculated using the number of observed ASVs and the inverse Simpson index, while beta diversity was estimated by computing Bray-Curtis distances between the genus-level profiles, which were then used as input for Principal Coordinates Analysis (PCoA).

All statistical analysis was performed in R (https://www.r-project.org/). PCoA plots were generated using the “vegan” (http://www.cran.r-project.org/package-vegan/) and “Made4” (Culhane et al., 2005) packages, and data separation was tested by a permutation test with pseudo-F ratio (function “Adonis” in “vegan”). For each PCoA plot, ellipses including 95% confidence area based on the standard error of the weighted average of sample coordinates were overlaid. The bacterial genera most contributing to the ordination space were identified using the function “envfit” of “vegan”. Linear discriminant analysis (LDA) effect size (LEfSe) algorithm with LDA score threshold of 3 (on a log10 scale) was applied on genus-level tables to identify discriminating taxa (Segata et al., 2011). Group differences in alpha diversity and taxon relative abundance were assessed by Wilcoxon test or Kruskal-Wallis test followed by post-hoc comparisons. P values were corrected for multiple comparisons using the Benjamini–Hochberg method. A false discovery rate (FDR) ≤ 0.05 was considered statistically significant. Sequences assigned to *Enterococcus* were aligned to the NCBI 16S rRNA database (September 2019 release) using BLASTn (Altschul et al., 1990); only hits with ≥80% identity were considered.

As for clinical data, categorical variables are presented as absolute numbers and relative frequencies; continuous variables are presented as mean and standard deviation if normally distributed or as median and IQR if non-normally distributed. The incidence rate of BSI due to *Enterococcus* spp. (*i.e.*, E-BSI) was calculated as the number of E-BSIs divided by the total number of 10,000 person-days at risk in ICU at St. Orsola Hospital in Bologna. The incidence rate was calculated for the first 4 months of 2020 (during the COVID-19 pandemic) and for the last three years over the same period (January-April). These values were compared by Poisson regression.

## Results

### Study Cohort Description

Of the 76 enrolled patients, 5 were excluded due to unavailable clinical data and 2 because of unconfirmed SARS-CoV-2 diagnosis, thus 69 were analyzed. Most of them were hospitalized at Bellaria hospital (N=51), 16 at Sant’Orsola and 2 at Maggiore hospital. The general characteristics of the study population are shown in **Table 1**. The median time from symptom onset to hospitalization was 3 days, 77% presented with a moderate/severe pneumonia according to respiratory parameters and imaging study, median of sequential organ failure assessment (SOFA) at hospitalization was 2. During hospitalization, 33% of patients developed severe respiratory failure, 23% were admitted to ICU and 14% were mechanically ventilated. Hydroxychloroquine and LMWH (low-molecular-weight heparin)-based treatments were administered in most patients (88.4%), while tocilizumab was used in 36% of enrolled patients. In-hospital death occurred in 9 patients (13%).

**Table 1:** Characteristics of study population.

	N = 69
**Demographics**	
Age (years) (median, IQR)	73, 59-85
Male	38 (55.1)
**Underlying diseases**	
Obesity	11 (15.9)
BMI (median, IQR)	24, 22-27
Hypertension	44 (63.8)
Diabetes mellitus	12 (17.6)
Coronary disease	5 (7.4)
Congestive heart failure	6 (8.8)
Cerebrovascular disease	12 (17.6)
Peripheral vascular disease	4 (5.9)
Chronic kidney disease	11 (15.9)
COPD	15 (22.1)
Immunosuppression	7 (10.1)
Charlson index (median, IQR)	5, 2-7
**Time from symptoms onset to hospitalization** (days) (median, IQR)	3, 1-7
**Symptoms at hospitalization**	
Fever ≥38°C	21 (30.4)
Cough	28 (40.6)
Dyspnoea	36 (52.2)
**Vital signs at hospitalization**	
GCS (median, IQR)	15, 15-15
MAP (median, IQR)	90, 81-100
PR (median, IQR)	87, 75-98
RR (median, IQR)	20, 16-25
SatO2 on ambient air (median, IQR)	96, 95-98
**Laboratory tests at hospitalization**	
Lymphocytes (10^9/L) (median, IQR)	1.06, 0.68-1.46
CRP (mg/dl) (median, IQR)	6.2, 2.7-13
LDH (IU/L) (median, IQR)	264, 213-379
**Imaging study**	
Lung consolidation	31 (44.9)
Ground glass	50 (72.5)
**COVID-19 severity at hospitalization**	
SOFA (median, IQR)	2, 1-3
**Therapeutic management of COVID-19**	
DRV/r	3 (4.4)
DRV/cobi	5 (7.2)
LMWH	58 (84.1)
Hydroxychloroquine	58 (84.1)
Tocilizumab	25 (36.2)
Steroids	1 (1.5)
**Supportive management of COVID-19**	
ICU admission	16 (23.2)
Mechanical ventilation	10 (14.5)
Inotropic support	7 (10.3)
Renal replacement therapy	4 (5.8)
ECMO	0
**Analysis of the gut microbiota**	
Time from hospitalization to fecal sample collection (days) (median, IQR)	14, 6-23
Exposure to antibiotics in the 2 weeks prior to fecal sampling	61 (88)
Bacterial infection after fecal sample collection	6 (8.7)
DTR infection after fecal sample collection	2 (2.9)
**Outcome**	
Severe respiratory failure	23 (33.3)
after collection of fecal sample	5 (7.2)
In-hospital mortality	9 (13)
Length of hospital stay (days) (median, IQR)	25, 13-33

BMI, body mass index; COPD, chronic obstructive pulmonary disease; CRP, C-reactive protein; DRV/cobi, darunavir/cobicistat; DRV/r, darunavir/ritonavir; DTR, difficult-to-treat resistance; ECMO, extracorporeal membrane oxygenation; GCS, Glasgow Coma Scale; IQR, interquartile range; LDH, lactate dehydrogenase; LMWH, low-molecular-weight heparin; MAP, mean arterial pressure; PR, pulse rate; RR, respiratory rate; SOFA, sequential organ failure assessment. Immunosuppression included neutropenia (neutrophil count <500/mm^3^), solid organ transplantation, hematopoietic stem cell transplantation, corticosteroid therapy at a dosage higher than or equivalent to prednisone 16 mg/day ≥15 days, uncontrolled HIV infection (<200 CD4/mm^3^). Unless otherwise specified, number and percentage (in brackets) are reported.

### The Gut Microbiota of COVID-19 Patients as Compared to Healthy Subjects

The GM of COVID-19 patients was profiled by 16S rRNA gene-based next-generation sequencing and compared with that of age- and sex-matched healthy Italians, whose sequences are publicly available (Biagi et al., 2016; De Filippis et al., 2016) (see *Materials and Methods*). A total of 2,792,602 reads (mean ± SD, 40,472 ± 11,243) were obtained and clustered into 12,889 Amplicon Sequence Variants (ASVs).

**Figure 1:**
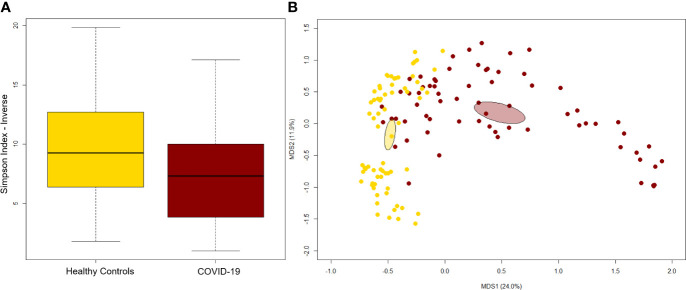
The gut microbiota of COVID-19 patients segregates from that of healthy subjects. **(A)** Alpha diversity estimated according to the inverse Simpson index. A significant reduction was observed in COVID-19 patients (p value = 0.0008, Wilcoxon test). **(B)** Principal Coordinates Analysis (PCoA) based on Bray-Curtis dissimilarity between the genus-level profiles. A significant separation between groups was found (p value = 0.001, permutation test with pseudo-F ratio). Ellipses include 95% confidence area based on the standard error of the weighted average of sample coordinates.

**Figure 2:**
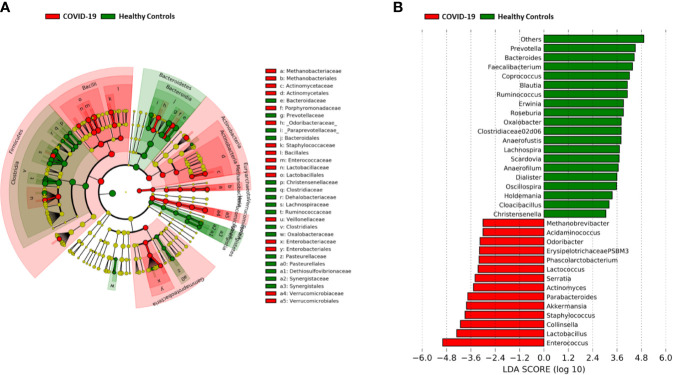
Gut microbiota signatures of COVID-19. **(A)** Cladogram of microbial taxa differentially represented between COVID-19 patients and healthy subjects at phylum to family level. The diameter of each circle is proportional to the taxon abundance. **(B)** Linear discriminant analysis (LDA) scores of discriminating genera between groups (the logarithmic threshold for discriminative features was set to 3.0). Plots were obtained by LDA effect size (LEfSe) analysis.

**Figure 3:**
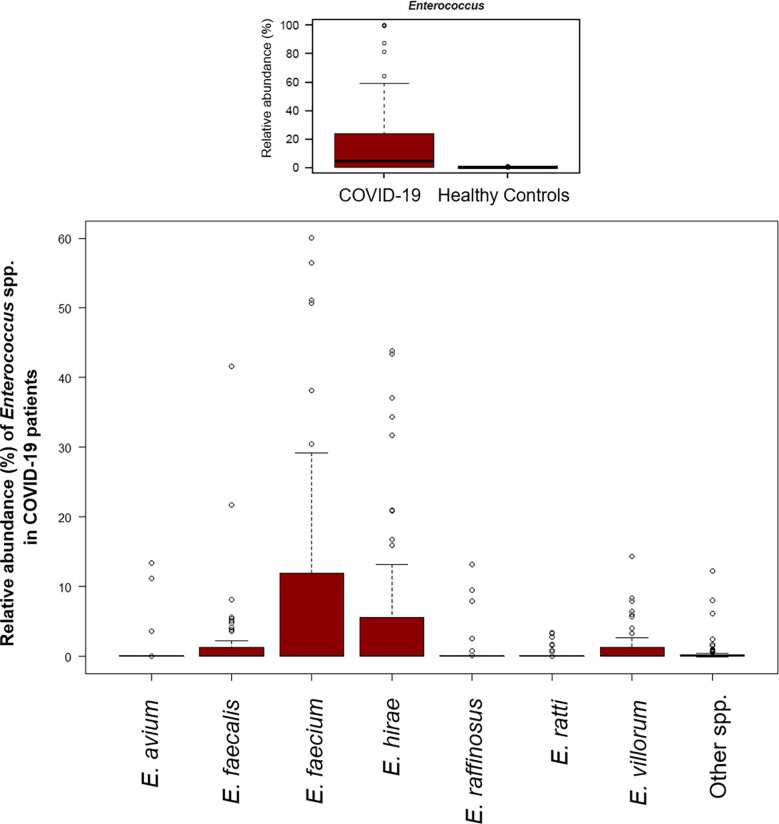
The gut microbial ecosystem of COVID-19 patients is enriched in *Enterococcus* spp. Boxplots showing the relative abundance distribution of the genus *Enterococcus* (upper panel) and its species (lower panel) in COVID-19 patients. Compared to healthy subjects, COVID-19 patients showed a significant overabundance of *Enterococcus* (p value ≤ 0.02, Wilcoxon test).

A significant reduction in alpha diversity was observed in COVID-19 patients compared to healthy controls (p value = 0.0008, Wilcoxon test) (**Figure 1A**). The PCoA of inter-individual variation, based on Bray-Curtis dissimilarity between the genus-level profiles, showed a significant separation between the groups (p value = 0.001, permutation test with pseudo-F ratio) (**Figure 1B**). As regards the composition, the GM of COVID-19 patients showed extensive destructuring even at high phylogenetic levels (see **Figure S1**). It was in fact characterized by alterations affecting the two dominant phyla, Firmicutes and Bacteroidetes, as well as subdominant components, mainly Actinobacteria and Synergistetes (p value ≤ 0.02, Wilcoxon test). At the family level, COVID-19 patients showed an enrichment in generally subdominant taxa, such as *Enterococcaceae*, *Coriobacteriaceae*, *Lactobacillaceae*, *Veillonellaceae*, *Porphyromonadaceae* and *Staphylococcaceae* (p value ≤ 0.03). On the other hand, their GM was characterized by a reduction of the dominant families *Bacteroidaceae*, *Lachnospiraceae* and *Ruminococcaceae*, as well as *Prevotellaceae* and *Clostridiaceae* (p value ≤ 0.001) (**Figures 2A** and **S1**). At the genus level (**Figure 2B**), as expected, taxa belonging to *Bacteroidaceae* (*i.e.*, *Prevotella* and *Bacteroides*), *Lachnospiraceae* (*i.e.*, *Coprococcus*, *Blautia*, *Roseburia* and *Lachnospira*) and *Ruminococcaceae* (*i.e.*, *Faecalibacterium*, *Ruminococcus*, *Oscillospira* and *Anaerofilum*) were the main discriminants of the GM of healthy subjects (p value ≤ 0.008). On the other hand, the GM of COVID-19 patients showed a distinctive pattern, with the enrichment of known or potential opportunistic pathogens, such as *Enterococcus*, *Staphylococcus*, *Serratia* and *Collinsella*, as well as *Lactobacillus*, *Parabacteroides*, *Lactococcus*, *Phascolarctobacterium*, *Odoribacter*, *Actinomyces*, *Methanobrevibacter* and *Akkermansia* (p value ≤ 0.02) (see **Figure S2**). According to a species-level analysis, the sequences assigned to *Enterococcus*, *i.e.*, the dominant genus in the GM of COVID-19 patients (mean relative abundance in COVID-19 patients vs healthy controls, 18.5% vs 0.05%), were mostly attributable to *E. faecium* (8.4%) along with *E. hirae* (5.5%), *E. faecalis* (1.8%) and *E. villorum* (1.1%) (**Figure 3**).

### Gut Microbiota Dysbiosis of COVID-19 Patients According to Intensive Care Unit Admission and Development of Bloodstream Infection

**Figure 4:**
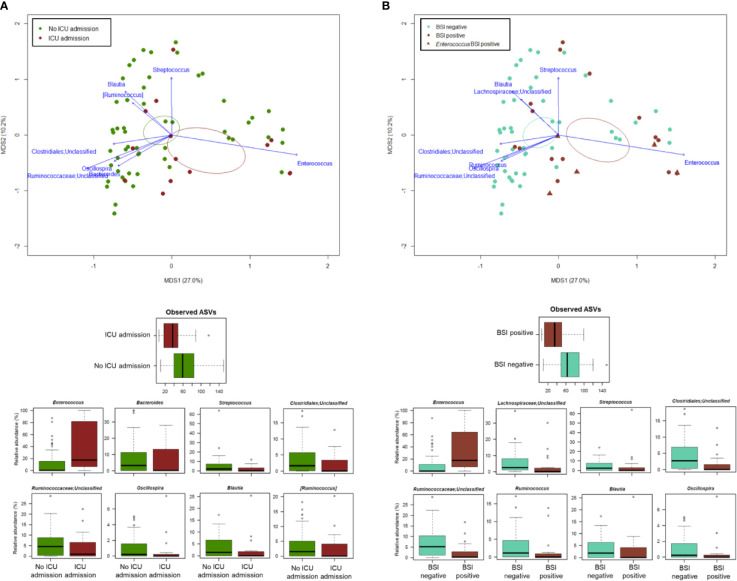
The gut microbiota dysbiosis of COVID-19 patients is exacerbated by ICU admission and development of BSI. Top, Principal Coordinates Analysis (PCoA) based on Bray-Curtis dissimilarity between the microbiota profiles of COVID-19 patients, stratified by ICU admission **(A)** and occurrence of BSI **(B)**. A significant separation was found for both variables (p value ≤ 0.05, permutation test with pseudo-F ratio). Ellipses include 95% confidence area based on the standard error of the weighted average of sample coordinates. Bacterial genera with the largest contribution to the ordination space are indicated with blue arrows (p value ≤ 0.001, permutational correlation test, “envfit” function). Bottom, Boxplots showing the distribution of alpha diversity, estimated according to the number of observed ASVs, and the relative abundance of genera differentially represented between groups (*i.e.*, COVID-19 patients admitted or not to the ICU, panel A; COVID-19 patients developing BSI or not, panel B) (p value ≤ 0.05, “envfit” function).

PCoA analysis based on Bray-Curtis dissimilarity between GM profiles of COVID-19 patients showed no segregation by age, sex, antibiotic intake in the 2 weeks prior to fecal sampling, length of hospital stay, time interval between fecal sampling and hospital admission, and outcome (death/discharge) (p value > 0.05, permutation test with pseudo-F ratio) (see **Figure S3**). On the other hand, the GM profiles were found to stratify by intensive care unit (ICU) admission and occurrence of bloodstream infection (BSI) (p value ≤ 0.05) (**Figure 4**). When looking for the bacterial genera driving the clustering patterns, we found that the same taxa were consistently depleted or enriched in relation to the two covariates (p value ≤ 0.001, “envfit” function). Specifically, ICU patients and those developing BSI were characterized by the over-representation (sometimes mono-dominance) of *Enterococcus* compared to the respective counterparts (p value ≤ 0.001). On the other hand, *Streptococcus*, *Oscillospira*, *Blautia* and other *Ruminococcaceae*, *Lachnospiraceae* and Clostridiales taxa were associated with COVID-19 patients who had not entered ICU and those who had not developed BSI (p value ≤ 0.001) (**Figure 4**). The severe destructuring of ICU and BSI-related GM profile was also evidenced by the further loss of alpha diversity (p value ≤ 0.004) (**Figure 4**). It should be noted that of the 16 COVID-19 patients who had entered ICU, 14 (87.5%) had developed BSI.

### The Gut Microbiota of Critically Ill COVID-19 *vs* Non-COVID-19 Patients

**Figure 5:**
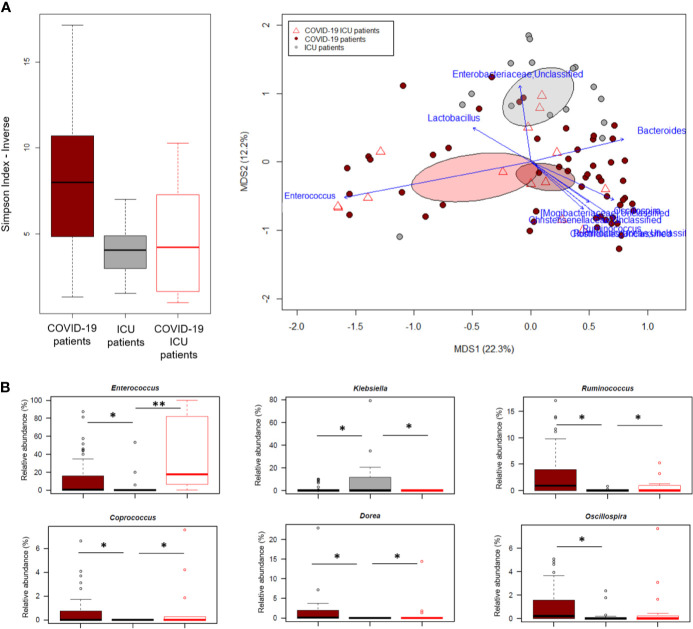
The gut microbiota dysbiosis of COVID-19 patients is distinct from that of critically ill non-COVID-19 patients. **(A)**, Left, Alpha diversity estimated according to the inverse Simpson index, for COVID-19 patients admitted or not to ICU and other critically ill patients admitted to ICU just before the COVID-19 outbreak. A significant reduction was observed in all patients admitted to ICU regardless of the underlying disease (p value = 0.0003, Kruskal-Wallis test). Right, Principal Coordinates Analysis (PCoA) based on Bray-Curtis dissimilarity between the genus-level profiles. A significant separation was found among the study groups (p value = 0.001, permutation test with pseudo-F ratio). Ellipses include 95% confidence area based on the standard error of the weighted average of sample coordinates. Bacterial genera with the largest contribution to the ordination space are indicated with blue arrows (p value ≤ 0.001, permutational correlation test, “envfit” function). **(B)**, Boxplots showing the relative abundance distribution of genera differentially represented between groups. *p value ≤ 0.05; **p value < 0.01; Wilcoxon test.

In an attempt to further explore the GM dysbiosis of COVID-19 patients, the sequences in this study were compared with those of critically ill non-COVID-19 patients who had been admitted to the ICU at St. Orsola hospital in October 2019-February 2020 following liver transplantation. Groups were best matched for known microbiota-associated confounding factors (*i.e.*, age and sex ratio) as well as for other therapy and hospitalization-related confounders (*i.e.*, antibiotic intake, time interval between hospitalization and fecal sampling, and development of bacterial infection) (see also Materials and Methods). Regardless of underlying disease, patients admitted to ICU showed comparable alpha diversity levels (p value > 0.05, Wilcoxon test), lower than COVID-19 patients who had not entered ICU (p value ≤ 0.004) (**Figure 5A**). In contrast, the GM structures of the three patient groups (*i.e.*, COVID-19 patients admitted or not to ICU and other ICU patients) clearly segregated in the Bray-Curtis-based PCoA space (p value = 0.001, permutation test with pseudo-F ratio). Several genera were found to drive this separation (p value ≤ 0.001, “envfit” function). Notably, *Enterococcus* was far overrepresented in both groups of COVID-19 patients compared to other ICU patients, but closely associated with COVID-19 patients admitted to ICU (p value = 0.0001, Wilcoxon test). Similarly, *Ruminococcus* was enriched in COVID-19 patients but associated with those who had not entered ICU (p value = 0.0003), as well as *Oscillospira*, *Dorea* and *Coprococcus* (p value ≤ 0.01). Critically ill non-COVID-19 patients were mainly discriminated by *Enterobacteriaceae* genera, particularly *Klebsiella* (p value ≤ 0.03) (**Figure 5B**).

### Incidence of Bloodstream Infection by *Enterococcus* spp. During the COVID-19 Outbreak

**Figure 6:**
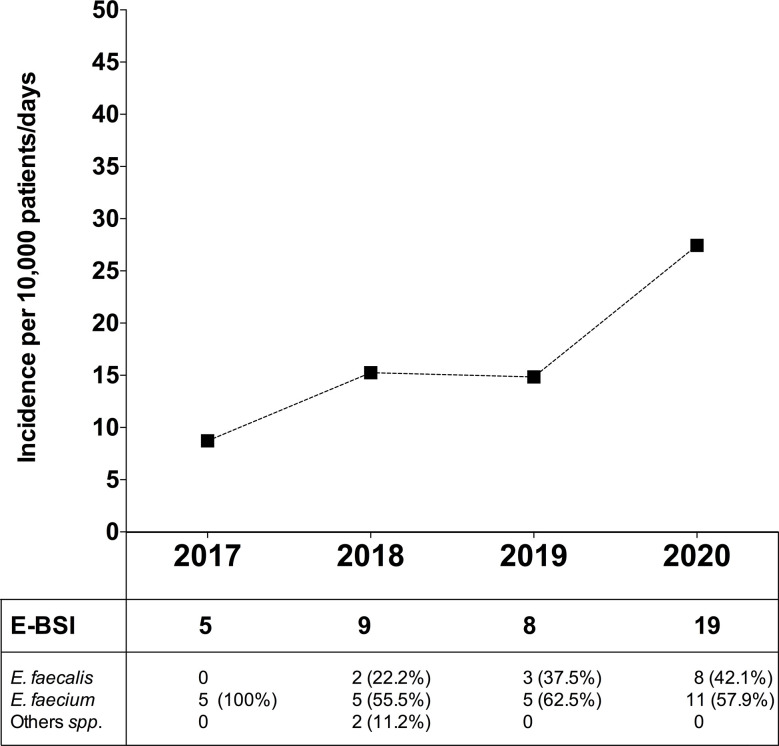
Incidence of bloodstream infection due to *Enterococcus* spp. in critically ill patients during the COVID-19 pandemic and in the previous 3 years. Top, Incidence rate of ICU-acquired enterococcal-BSI (E-BSI) per 10,000 patient-days during the COVID-19 pandemic and in the previous 3 years. The incidence rate was evaluated over the same 4-month period (January-April) since 2017 to 2020. Bottom, Number of cases of BSI due to *E. faecium*, *E. faecalis* and others (*Enterococcus* spp.) over the studied periods.

In order to evaluate the dynamic change in BSI due to *Enterococcus* spp. (E-BSI) during the COVID-19 pandemic, we compared the E-BSI incidence in critically ill patients over the same period (January-April) since 2017 to 2020 (**Figure 6**). During the first 4 months of 2020, 1,317 patients were admitted to the ICU with a mean length of stay of 5.3 days and a total patient days of 6,924. The incidence rate of ICU-acquired E-BSI reached 27.4 (95% CI, 1.75-4.29) per 10,000 patient-days during the COVID-19 outbreak. In detail, we observed 19 episodes of E-BSI in the first 4 months of 2020 among patients recovered in ICU, mainly due to *E. faecium* (57.9%). A significant increase in the incidence rate of E-BSI was observed between 2017 and 2020 (p-value = 0.01, Poisson regression). This incidence rate was 14.8 (95% CI, 0.74–2.96) in 2019 and 15.2 (95% CI, 0.79–2.92) in 2018. The relative risk of ICU-acquired E-BSI during the first 4 months of 2020 was 1.84/3.14-fold higher than in previous years.

## Discussion

We profiled the GM of 69 Italian patients affected by COVID-19 during the first wave in Italy. Consistent with previous reports on relatively small cohorts of Chinese patients (Gu et al., 2020; Zuo et al., 2020a; Chen et al., 2021; Zuo et al., 2021), their GM appears severely dysbiotic, with distinct signatures compared to healthy subjects. In addition to a loss of diversity, COVID-19 patients show profound GM destruction, with drastic reduction in the relative abundance of the dominant families *Bacteroidaceae*, *Lachnospiraceae* and *Ruminococcaceae*, well known to be associated with health and to produce SCFAs, *i.e.*, microbial metabolites with a key, multifaceted role in human metabolic and immunological homeostasis (Koh et al., 2016). On the other hand, we found increased proportions of potential pathobionts, mostly belonging to *Enterococcaceae*, *Coriobacteriaceae* and *Staphylococcaceae*. Among these, it is worth noting the presence of *Collinsella* and *Actinomyces*, both recently found enriched in fecal samples from Chinese COVID-19 patients, with the latter supposed to derive from extra-intestinal sites, such as the oral cavity or upper respiratory tract (Gu et al., 2020; Zuo et al., 2020a; Zuo et al., 2021). Although some of the aforementioned microbial traits are common to other disorders, both intestinal and systemic (Duvallet et al., 2017), the remarkable enrichment of *Enterococcus* seems to represent a distinctive GM footprint of our cohort. In some patients, GM was even almost mono-dominated by *Enterococcus* spp., mostly *E. faecium*, *E. hirae*, *E. faecalis* and *E. villorum*. It is known that a high abundance of *Enterococcus* in the GM of critically ill patients may be clinically relevant given its pathogenic potential, intrinsic resistance to many commonly used antimicrobials, and the ability to rapidly acquire resistance determinants against virtually all antibiotics (Gilmore et al., 2014). Regardless of the source of enterococcal strains, the GM of COVID-19 patients may therefore act as a reservoir of opportunistic, potentially antibiotic-resistant pathogens, with he potential to translocate across compromised epithelial barriers into circulation, as already demonstrated in other disease contexts (Tamburini et al., 2018; Agudelo-Ochoa et al., 2020).

The severity of COVID-19-related dysbiosis was found to be strongly associated with development of BSI and ICU admission. In particular, the GM of BSI positive and ICU COVID-19 patients was even less diverse, and showed a further increase in *Enterococcus* along with a reduction in *Ruminococcaceae* and *Lachnospiraceae* taxa. Furthermore, patients admitted to ICU showed a depletion of *Bacteroides*. Interestingly, *Bacteroides* species have been shown to negatively correlate with the fecal load of SARS-CoV-2 in COVID-19 patients and suggested to be involved in the regulation of ACE2 expression (Zuo et al., 2020a). In an attempt to further explore the impact of ICU stay, we compared the GM of COVID-19 patients with that of patients admitted to the ICU just before the COVID-19 outbreak. According to our findings, *Enterococcus* was far overrepresented in the GM of COVID-19 patients, especially those admitted to ICU, while almost absent in critically ill non-COVID-19 patients. Conversely, the latter were discriminated by higher proportions of *Enterobacteriaceae* members, especially *Klebsiella*. Although we are aware that we cannot claim that the high proportions of enterococci are specific to COVID-19, it is interesting to note that the percentage of patients who developed E-BSI was significantly higher during the COVID-19 pandemic than in the previous 3 years. On the other hand, recent studies have shown a similar increase in E-BSI in critically ill COVID-19 patients in several European (Italian and Spanish) hospitals (Bardi et al., 2021; Bonazzetti et al., 2021), suggesting that the development of secondary infections by *Enterococcus* in these patients could be a general healthcare problem, somehow related to SARS-CoV-2 infection.

Several limitations of this study should be mentioned: small sample size, stool collection at different time intervals, non-standardized bacterial superinfection diagnosis and therapy protocols, hospitalization in several ICUs (however close to each other), the lack of a non-COVID-19 control group from the same hospitals at the same time, and multiple GM-associated confounders, including antibiotic intake, which may have partially biased our findings. Moreover, due to the cross-sectional nature of our study, we could not provide information on the temporal dynamics of GM during SARS-CoV-2 infection and recovery. In this regard, Chen and colleagues have recently characterized the GM trajectory in COVID-19 patients from diagnosis up to 6 months after hospital discharge, highlighting that the dramatic loss of GM richness persisted during recovery and was associated with worse pulmonary functions (Chen et al., 2021).

In conclusion, while confirming the existence of severe GM dysbiosis in COVID-19 patients, our work highlights a peculiar overrepresentation of *Enterococcus*, closely related to ICU admission and development of BSI. As recently discussed, (Agudelo-Ochoa et al., 2020; Chen et al., 2021), GM monitoring in critically ill patients, including COVID-19 ones, could help clinical management, by predicting the onset of medical complications such as sepsis and mortality, thus allowing timely adoption of countermeasures aimed at alleviating the already weakened condition of these subjects and speeding up their recovery. Future studies in independent and much larger cohorts from varied geographical contexts, possibly with multi-omics approaches, are needed to validate our findings, and deepen GM-host relationships and their contribution to the disease course. In particular, longitudinal studies are warranted for a fine resolution of GM changes even in the long term. In this regard, the ongoing EU project ORCHESTRA foresees a prospective follow-up for SARS-CoV-2 infected individuals, with the aim of exploring the long-term consequences of COVID-19.

## Data Availability Statement

Sequencing reads were deposited in theNational Center for Biotechnology Information Sequence Read Archive (NCBI SRA; BioProject ID PRJNA700830).

## Ethics Statement

The study was approved by Comitato Etico Indipendente di Area Vasta Emilia Centro, n. 283/2020/Oss/AOUBo. The patients provided their written informed consent to participate in this study.

## Author Contributions

Conceptualization, PB, MR, AS, and PV. Formal analysis, FD’A, ST, SR, PG, and DL. Data curation, MG, MB, GF, and SC. Writing-original draft preparation, FD’A, ST, PG, and MG. Writing-review and editing, PB and MB. Visualization, FD’A and PG. Supervision, PB, MR, and PV. Project administration, PB, MR, and PV. All authors contributed to the article and approved the submitted version.

## Conflict of Interest

The authors declare that the research was conducted in the absence of any commercial or financial relationships that could be construed as a potential conflict of interest.
