# Supplementary material for: The Gut Microbiota of Critically Ill Patients With COVID-19
Source: Front Cell Infect Microbiol. 2021 Jun 29;11:670424. doi: 10.3389/fcimb.2021.670424 (PMC8276076; doi:10.3389/fcimb.2021.670424)
Supplement: Supplementary file 1 [file DataSheet_1.pdf]

Supplementary Material

Supplementary Methods

Total microbial DNA was extracted from fecal samples by using the repeated bead-beating plus column method, as previously described with few modifications (Turroni et al., 2017). Briefly, 250 mg of stool sample were resuspended in 1 mL of lysis buffer (500 mM NaCl, 50 mM Tris-HCl pH 8, 50 mM EDTA, 4% (w/v) SDS) with four 3-mm glass beads and 0.5 g of 0.1-mm zirconia beads (BioSpec Products), and homogenized using a FastPrep instrument (MP Biomedicals) at 5.5 movements/sec for 1 min, repeated three times. After incubation at 95°C for 15 min, stool particles were pelleted by centrifugation at 13,000 rpm for 5 min. The supernatant was added with 260 µL of 10 M ammonium acetate and, after 10-minute centrifugation at 13,000 rpm, incubated with one volume of isopropanol in ice for 30 min. Nucleic acids were precipitated by 15-minute centrifugation at 13,000 rpm and washed with 70% ethanol, then resuspended in TE buffer (10 mM Tris-HCl, 1 mM EDTA pH 8.0). After treatment with 2 µL of 10 mg/mL DNase-free RNase at 37°C for 15 min, samples were subjected to protein removal and column-based DNA purification following the manufacturer's instructions (DNeasy Blood and Tissue Kit; QIAGEN). DNA concentration and quality were assessed with the NanoDrop ND-100 spectrophotometer (NanoDrop Technologies). For library preparation, the V3-V4 hypervariable region of the 16S rRNA gene was amplified by using the 341F and 785R primers with added Illumina adapter overhang sequences, as previously described (D'Amico et al., 2019). Amplification was performed using KAPA HiFi HotStart ReadyMix (Roche), and programming a Thermal Cycler T (Biometra) as follows: 3 minutes at 95°C, 25 cycles of 30 seconds at 95°C, 30 seconds at 55°C, and 30 seconds at 72°C, and a final 5-minute step at 72°C. PCR products were purified using a magnetic bead-based clean-up system (Agencourt AMPure XP; Beckman Coulter). A limited-cycle PCR was performed to obtain the indexed library using Nextera technology, followed by a second AMPure XP magnetic bead-based purification. Final libraries were pooled at equimolar concentration (4 nM), denatured with 0.2 N NaOH, and diluted to 5 pM with a 20% PhiX control before sequencing on an Illumina MiSeq platform, with a 2 × 250 bp paired-end protocol according to the manufacturer's instructions (Illumina).

References

D'Amico, F., Biagi, E., Rampelli, S., Fiori, J., Zama, D., Soverini, M., et al. (2019). Enteral nutrition in pediatric patients undergoing hematopoietic SCT promotes the recovery of gut microbiome homeostasis. *Nutrients.* 11:2958. doi: 10.3390/nu11122958

Turroni, S., Rampelli, S., Biagi, E., Consolandi, C., Severgnini, M., Peano, C., et al. (2017). Temporal dynamics of the gut microbiota in people sharing a confined environment, a 520-day ground-based space simulation, MARS500. *Microbiome*. 5:39. doi: 10.1186/s40168-017-0256-8

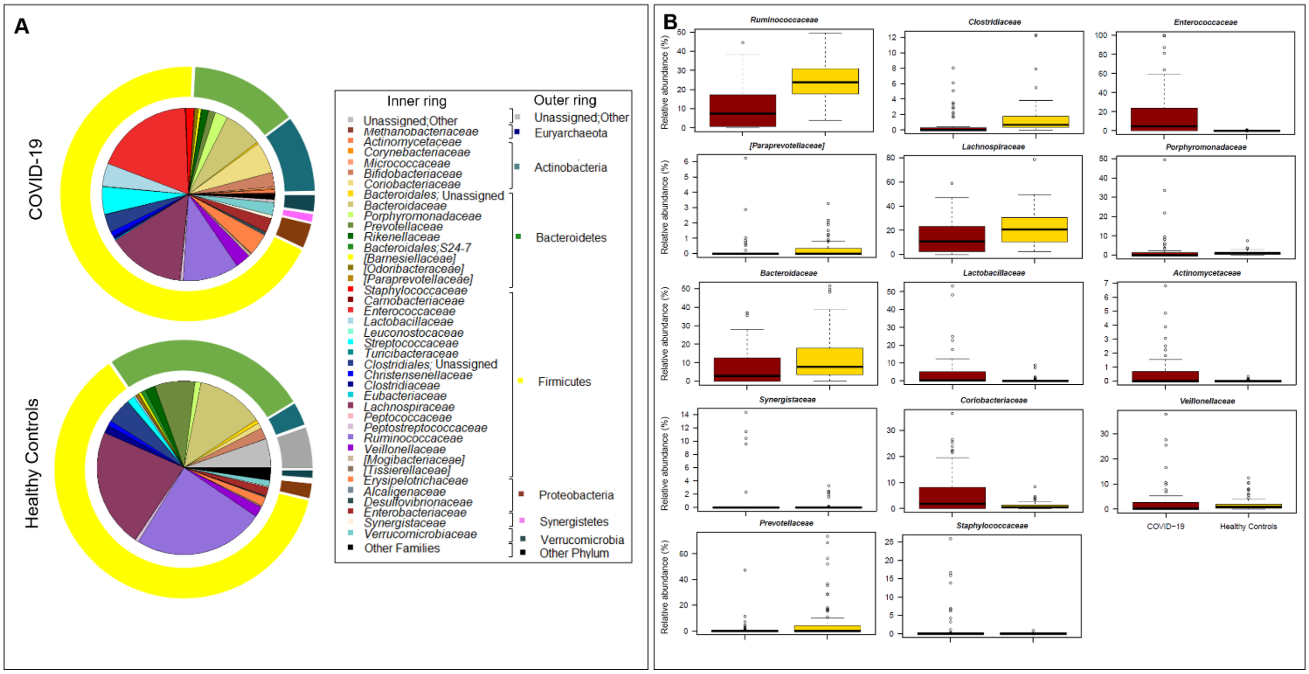

Figure S1. Phylum and family-level structure of the gut microbiota of COVID-19 patients.

A, Pie charts showing the relative abundance of the major families in the gut microbiota of COVID-19 patients and healthy subjects. Outer rings depict phylum-level distribution. Only taxa with relative abundance >0.1% in at least 2 samples were considered. **B**, Boxplots showing the relative abundance distribution of families differentially represented between groups (p value $\leq$ 0.05, Wilcoxon test).

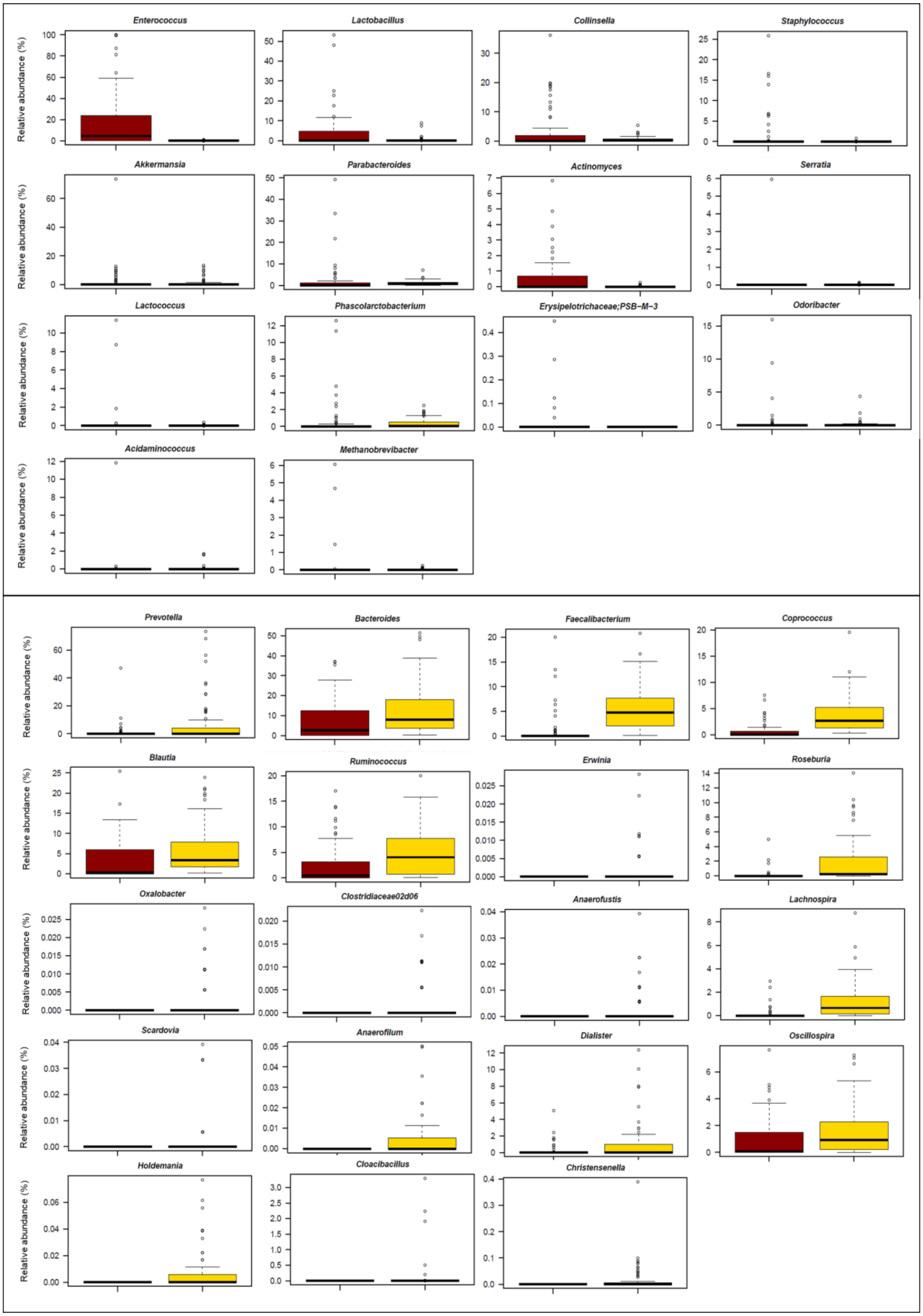
Enterococcus
Lactobacillus
Collinsella
Staphylococcus
Akkermansia
Parabacteroides
Actinomyces
Serratia
Lactococcus
Phascolarctobacterium
Erysipelotrichaceae;PSB-M-3
Odoribacter
Acidaminococcus
Methanobrevibacter
Prevotella
Bacteroides
Faecalibacterium
Coprococcus
Blautia
Ruminococcus
Erwinia
Roseburia
Oxalobacter
Clostridiaceae02d06
Anaerofustis
Lachnospira
Scardovia
Anaerofilum
Dialister
Oscillospira
Holdemania
Cloacibacillus
Christensenella
Relative abundance (%)

Figure S2. Bacterial genera differentially represented between COVID-19 patients and healthy controls.

Boxplots showing the relative abundance distribution of the genera differentially represented between groups according to the LEfSe algorithm with LDA score threshold of 3 (on a log10 scale). Red, COVID-19 patients; yellow, healthy controls.

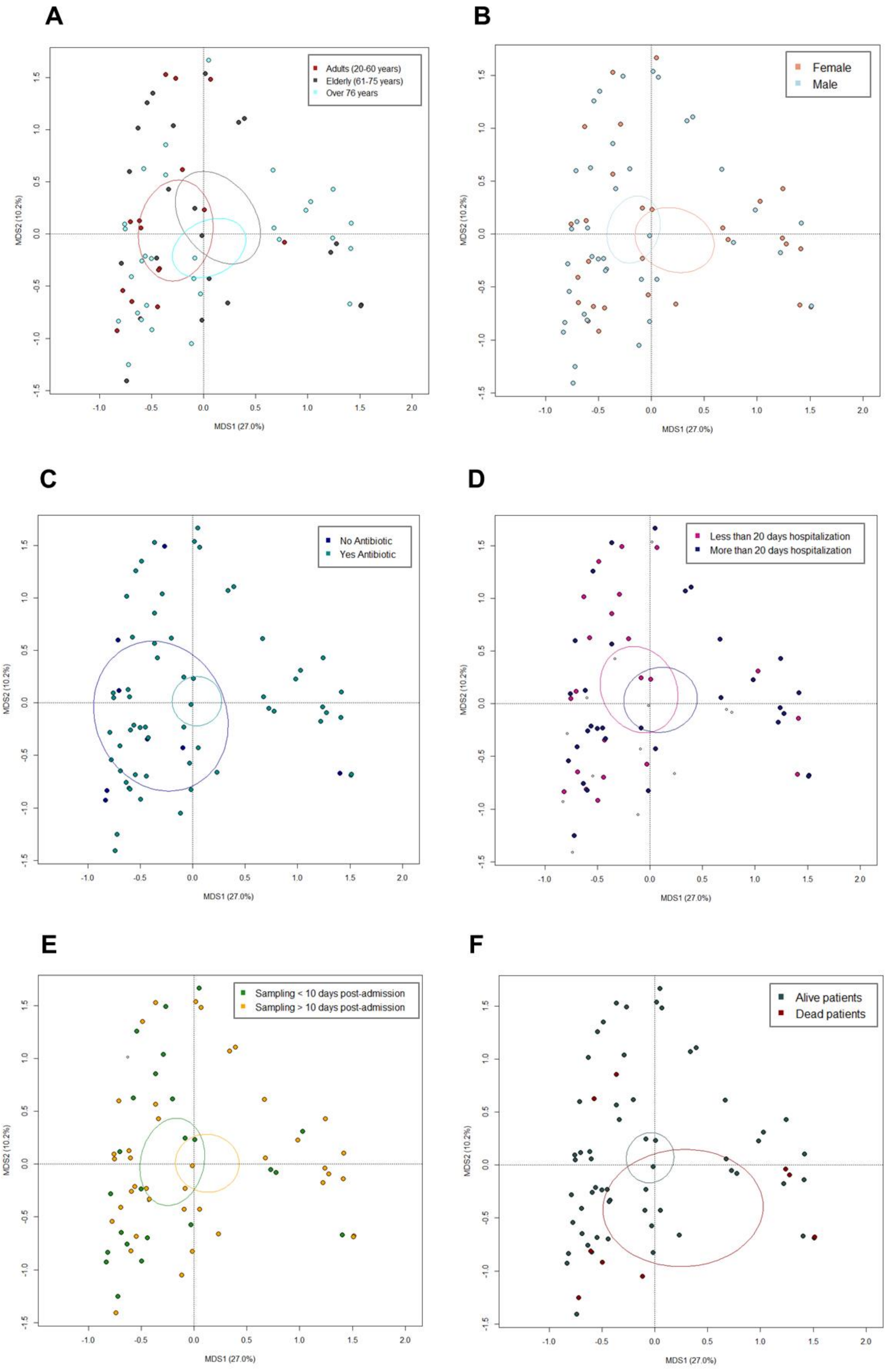
A
Adults (20-60 years)
Elderly (61-75 years)
Over 76 years
MDS1 (27.0%)
MDS2 (10.2%)
B
Female
Male
MDS1 (27.0%)
MDS2 (10.2%)
C
No Antibiotic
Yes Antibiotic
MDS1 (27.0%)
MDS2 (10.2%)
D
Less than 20 days hospitalization
More than 20 days hospitalization
MDS1 (27.0%)
MDS2 (10.2%)
E
Sampling < 10 days post-admission
Sampling > 10 days post-admission
MDS1 (27.0%)
MDS2 (10.2%)
F
Alive patients
Dead patients
MDS1 (27.0%)
MDS2 (10.2%)

Figure S3. Gut microbiota dysbiosis of COVID-19 patients is robust to several confounding factors.

Principal Coordinates Analysis (PCoA) based on Bray-Curtis dissimilarity between fecal samples from COVID-19 patients stratified by age group (adults, elderly and over 76 years, **A**), gender (males vs females, **B**), antibiotic intake in the 2 weeks prior to fecal sampling (yes vs no, **C**), length of hospital stay (more or less than 20 days, **D**), time interval between fecal sampling and hospital admission (more or less than 10 days, **E**) and final outcome (dead vs alive patients, **F**). Ellipses include 95% confidence area based on the standard error of the weighted average of sample coordinates. No significant separation was found (p value > 0.05, permutation test with pseudo-F ratio).
